# Go with the flow: Impacts of high and low flow conditions on freshwater mussel assemblages and distribution

**DOI:** 10.1371/journal.pone.0296861

**Published:** 2024-02-15

**Authors:** Kiara C. Cushway, Aubrey E. Harris, Candice D. Piercy, Zachary A. Mitchell, Astrid N. Schwalb

**Affiliations:** 1 Department of Biology, Texas State University, San Marcos, Texas, United States of America; 2 Engineer Research and Development Center, United States Army Corps of Engineers, Vicksburg, Mississippi, United States of America; Marine Science Centre, University of Basrah, IRAQ

## Abstract

Understanding the drivers of distribution and assemblage composition of aquatic organisms is an important aspect of management and conservation, especially in freshwater systems that are inordinately facing increasing anthropogenic pressures and decreasing biodiversity. For stream organisms, habitat conditions during high flows may be impossible to measure in the field, but can be an important factor for their distribution, especially for less mobile organisms like freshwater mussels. Hence, the objective of this study was to use a two dimensional HEC-RAS model to simulate hydraulic conditions during high and baseline flows (flows approx. 10–600 x and 0.7 x median daily flows respectively) in a 20 km segment in the San Saba River, Texas in combination with existing mussel survey data from 200 sites (collected every 100m) to 1) examine whether hydraulic conditions differed between areas of increased mussel richness and diversity (referred to as hotspots) and other sites, and 2) understand how well site occupancy and species abundance could be explained by hydraulic conditions occurring under different flow conditions. The results showed that richness and diversity hotspots occurred in deeper areas with lower shear stress, stream power, and Froude number during both high and low flows. Occupancy could be predicted with 67–79% accuracy at the site scale and 60–70% accuracy at the mesohabitat scale (∼20 to 1200 m long). In addition, hydraulic conditions across flow scenarios explained up to 55% of variation in species abundances, but predictions were less successful for species often observed to occupy micro-scale flow refuges such as bedrock crevices. The results indicate that pools may serve as important refuge for all species during both high and low flow events, which may be relatively unique to bedrock-dominated systems. Understanding hydraulic conditions that occur at extreme flows such as these is important given that the frequency and magnitude of such events are increasing due to climate change.

## Introduction

Freshwater ecosystems host a broad diversity of organisms and provide essential services to both organisms and society but are inordinately imperiled [1]. In addition, understanding distribution patterns is an important aspect for effective conservation and management strategies. The factors that influence distribution patterns of species operate at multiple spatial and temporal scales and include rare events that can be important for structuring communities [2–4]. Thus, the patchy distribution of organisms may be the result of conditions that cannot be sampled directly, as they may have occurred during rare or otherwise challenging to survey events. Modeling of conditions during these rare events can thus fill an important knowledge gap.

Organisms in the order Unionida, hereafter termed ‘mussels,’ are a useful study organism for understanding how rare events may affect distribution because they can be relatively long-lived (sometimes > 40 years) and primarily sedentary organisms that have limited dispersal ability, relying primarily on host fish for large scale dispersal. Mussels are globally distributed, provide important ecosystem services like water filtration, biodeposition, and habitat stabilization [5, 6], and are highly imperiled [7, 8]. Mussels are threatened by various disturbances including altered flow regimes, pollution, habitat destruction, and climate change, particularly the increase in magnitude and frequency of extreme high and low flow events [8–10].

Mussels have a limited ability to escape or recover from unfavorable flow conditions and may be affected by greatly varying flows during their lifespan [11]. High flows (i.e., flows higher than the average flow) and flood events (i.e., high flows during which the riverbanks are overflown) can alter available habitat and lead to population and community level impacts [12–14]. Flooding has led to massive losses in mussel populations (*e*.*g*., >50,000 *Margaritifera margaritifera* in a single event in Scotland) [12]. Furthermore, floods may impact population dynamics of mussels by reducing abundance, survivorship, and site fidelity [13]. Shifts in distribution and community composition in response to flooding have been documented [14], and distribution of mussels is often associated with flow refuges [11, 15, 16]. The mechanisms by which flooding and high flow events affect freshwater mussels are frequently related to sediment and bed mobility [11, 16]. High shear stress and stream power associated with high flows may mobilize substrate and dislodge mussels, potentially transporting them to unfavorable habitats [17–19]. Mussels may become stranded in areas that are inundated during flooding but dry out as water levels recede [12, 20]. In addition, mussels can be damaged or crushed by larger substrates mobilized during flood events [12]. In hydraulics, such turbulence and flow characteristics are quantified by dimensionless parameters like Froude number and Reynold’s number. These parameters are shown to be related to bed mobility during high flows [21, 22].

At low flows, mussels may be subjected to low dissolved oxygen levels and increased water temperatures, especially during periods of drought [23, 24]. Mussels are filter feeders, and feeding and clearance rates tend to increase with increasing flow [25], so low flows may impact mussel survival if food delivery is decreased. Depth plays an important role in maintaining suitable wetted habitat for mussels during low flows, although studies examining the influence of water depth on mussel mortality, presence, and abundance have generated mixed results [26]. Habitat stability during low flow conditions is also important to mussel populations [27], although some studies indicate that conditions during high flows may be more influential [17].

Simple hydraulic variables such as water velocity and depth characterize the flow rate and volume of water in a system and can be directly measured [26], while complex hydraulic variables like shear stress, Froude number, and stream power combine multiple simple hydraulic variables to explain more complicated flow patterns that influence the hydraulic conditions organisms are exposed to in a system [26]. While there is generally a good understanding of how both simple and complex hydraulic variables influence mussel presence and abundance in streams [17, 19, 28], less is known about how such variables influence species richness and diversity [26]. Because mussels may exhibit species-specific responses to hydraulic conditions [26], individual species may be differentially impacted by flow conditions, resulting in potentially complex impacts on both the number of species occurring in a particular habitat and the composition of distinct assemblages that may be missed if only presence or abundance of mussels are examined. Furthermore, to the best of our knowledge, there is only one previous study regarding the impact of hydraulic conditions on mussels in bedrock dominated streams, where substrate tends to be more stable [15] (Table 1).

**Table 1:** Studies examining the impacts of hydraulic conditions on freshwater mussels at different flows in variable environments at multiple spatial scales.

Dominant substrate	Study	Scale	Flow	Response variables	Model used	River
**Bedrock**	This study	Segment	L, MO, H	Presence, species richness, Shannon’s/Simpson’s diversity, *L*. *bracteata*, *U*. *imbecillis*, and *C*. *tampicoensis* abundance	2-D HEC-RAS	San Saba River, TX, USA
	[15]	Segment	L, H	Presence, distribution, abundance	1D HEC-RAS	South Fork Eel River, CA and USA
**Cobble**	[29]	Catchment, reach, channel unit	L, M, H	Presence, distribution, habitat suitability	SWAT; 1D HEC-RAS	Aist Catchment, AT
**Cobble-gravel**	[16]	Reach	L, H	Presence, predicted habitat	FaSTMECH	Trinity River, CA, USA
	[27]	Reach	L, MO, H	Abundance, distribution	IFG4, MANSQ	Horse Lick Creek, KY, USA
**Cobble-sand**	[11]	Reach (multi-river)	L, H	Abundance, presence, tracer movement	NA	Neversink River and Webatuck Creek, NY, USA
	[30]	Reach (multi-river)	L, M, H	NA	1D HEC-RAS	Feldaist, Kamp, Maltsch, Waldaist, Rodl, Aist, and Gusen Rivers, AT
**Cobble-gravel-silt**	[31]	Reach	L	Abundance, distribution	NA	Allegheny River, PA, USA
	[32]	Segment, reach	L	Abundance	NA	Allegheny River, PA, USA
**Gravel**	[17]	Segment	L, H	Species richness, abundance	NA	Little River, Oklahoma, USA
	[33]	Basin	L, B	Juvenile mortality	1D HEC-RAS	Danube River Basin, AT
**Gravel-clay**	[28]	Segment	L, M. H	Presence, abundance	NA	Mississippi River, MN and WI, USA
**Pebble-sand**	[34]	Multi-river	BF, B	Mussel bed persistence, catch per unit effort, species richness	Nays2DH	Tonawanda and French Creeks, NY, USA
**Sand**	[19]	Segment	L, H	Abundance (*Castalia ambigua* and *Anodontites elongatus*)	NA	Amazon River, Pará, BR
	[35]	Segment	B	Abundance	NA	Apalachicola River, FL, USA
	[36]	Reach (multi-river)	L, H	Abundance, species richness, presence	NA	Brazos and Trinity Rivers, TX, USA
**Sand-silt**	[37]	Segment, reach	M	Presence, abundance, Shannon’s diversity index	3D Delft model	Saint John River, NB, CA
**Alluvial**	[38]	Multi-scale (macro, meso, micro)	L	Presence (*Alasmidonta heterodon*), habitat suitability	River2D, MesoHABSIM	Upper Delaware River, NY, NJ, PA, USA
**Alluvial-lacustrine**	[39]	Basin, channel unit	L	Abundance, distribution	NA	Klamath River, OR, USA
**Fine to large substrate**	[40]	Multi-scale (basin, reach, channel unit, microhabitat)	BF, B	Catch per unit effort (CPUE), species richness, indicator species	STREAM	Neches River, TX, USA
**Not specified**	[4]	Multi-scale (sub-watershed, segment, channel unit, reach)	BF	Abundance, distribution, species abundance	NA	Middle Fork John Dy River, OR, USA
	[41]	Catchment	L, B	Presence	HYDRO_AS-2D	Danube River Basin, Bavarian Forest, DE
	[42]	Catchment	BF, B	Catch per unit effort (CPUE), species richness	NA	Coosa River, USA
	[43]	Multi-basin	L, H	Presence (*Pleurobema riddelli*, *Potamilus amphicaenus*, and *Truncilla macrodon*)	IHA	Sabine, Trinity, Neches, and Cypress Rivers, TX, USA
	[44]	Multi-river	MO	Abundance	NA	Green, Licking, and Rough Rivers, KY, USA
	[45]	Multi-segment	L, M, MO, H	Juvenile dispersal	CFD model	Upper Mississippi River, MO, USA
	[46]	Segment	L, M, MO, H	Juvenile settlement	FLUENT	Upper Mississippi River, MO, USA
	[47]	Segment	B	Presence (*Tritogonia verrucosa* and *L*. *teres*)	STREAM	Sabine River, TX, USA
	[48]	Segment	L	Abundance, species richness, presence (*L*. *higginsii* and *Quadrula fragosa*)	NA	St. Croix River, WI, USA
	[49]	Segment	L, H	Abundance, presence, various species abundances	NA	Upper Mississippi River, WI, USA

L = low flow, BF = baseflow, M = mean or median flow, MO = moderate flow, B = bankfull flow, H = high flow

The objective of this study was to use hydraulic modeling techniques to understand how hydraulic conditions during high and low flow events influence freshwater mussel communities in a bedrock-dominated river system. Hydraulic modeling is a useful tool for simulating physical properties of rivers and can be used to infer how flow interacts with the landscape to produce conditions experienced by in-stream organisms. Two-dimensional hydraulic models provide a means to quantify biologically important flow conditions at relevant spatial resolutions that may be oversimplified in more widely used one-dimensional models [50]. Furthermore, hydraulic modeling permits the examination of hydraulic conditions during extreme flows when field sampling is unsafe or infeasible. In this study, a two-dimensional, unsteady flow model was used to simulate a low flow and flows with 10% and 50% exceedance probabilities. These simulations were used to answer the following questions using data collected at the segment scale:

1. Do hydraulic conditions at high (approx. 10–600 x median daily flows) and baseline low (approx. 0.7 x median daily flows) flows differ between hotspots of mussel richness and diversity and other sites?
2. Can site occupancy and species abundances be accurately predicted based on different hydraulic conditions occurring during high and low flows?

Our results shed light on the importance of hydraulic habitat conditions on mussel distribution and assemblages, especially in understudied bedrock systems.

## Materials and methods

### Study area

**Fig 1:**
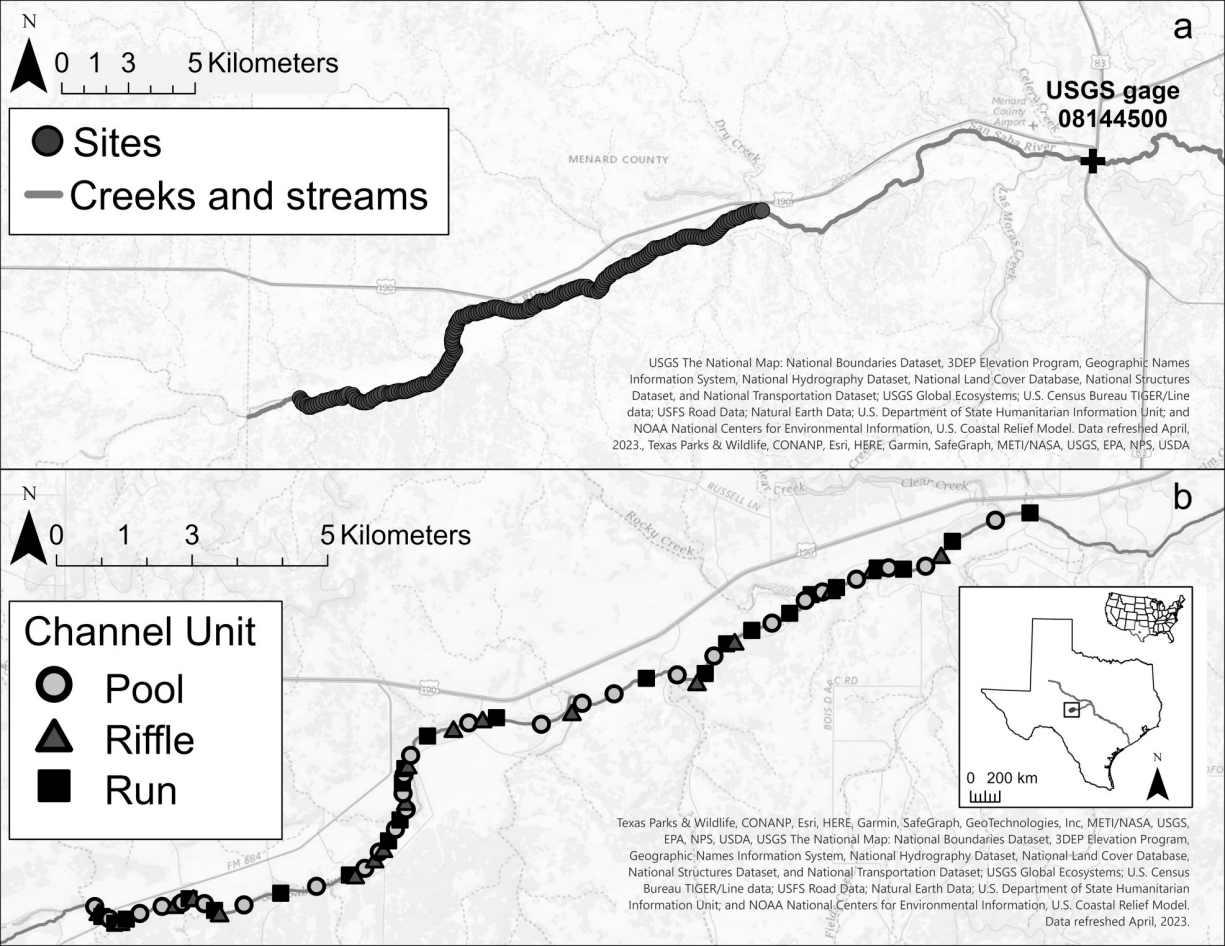
Location of A) 200 study sites and B) 70 mesohabitat units in the San Saba River, TX and USGS gage 08144500 used for model construction. Information about mussel assemblages and habitat characteristics necessary for HEC-RAS model construction were collected at each site. Notice difference in scale. Map services and data available from U.S. Geological Survey, National Geospatial Program.

The San Saba River is a tributary of the Colorado River that runs through central Texas, which is considered one of the most flash-flood prone regions of the United States [51]. The San Saba River flows along the north edge of the Edwards Plateau region and is predominantly surrounded by ranch or agricultural land and shrubland with prevalent limestone [52]. Annual rainfall averages between 55.9 and 86.4 cm [53], with the flow regime consisting predominantly of low flow periods interrupted by short-term but high-intensity high flow events [54]. The upper section of the San Saba River (above Menard, Texas) is spring fed by several natural springs, including Fort McKavett and Clear Creek Springs [55]. Portions of the San Saba River, including the upper, and more commonly, the middle segments have been characterized as intermittent due to anthropogenic water use [55–57]. This study was conducted at 200 sites in a 20 km stretch of the San Saba River between Fort McKavett and Menard, Texas (Fig 1). Survey sites occurred in pool, riffle, or run mesohabitats (*i*.*e*., distinct morphological units that differ in hydraulic conditions). Median daily discharge for the study segment was approximately 0.62 m^3^s^-1^ between 1916 and 2018 (USGS gage 08144500) [58].

Across the 200 sampling sites in our study area, which occurred in 70 distinct mesohabitat units (*i*.*e*., pools, riffles, or runs), a total of 859 mussels of nine species were found (Table 2) [59]. Live mussels were found at 104 sites (52%) in 35 mesohabitat units (50%), and the maximum number of species found at any given site or mesohabitat was six, with an average of 0.89 species p-h^-1^ per site and 1.04 species p-h^-1^ per mesohabitat unit. The most abundant species were *Lampsilis bracteata*, followed by *Utterbackia imbecillis* and *Cyrtonaias tampicoensis*. Less than 10 individuals of *Pustulosa pustulosa* and *Fusconaia iheringi* were found [59].

**Table 2:** Abundance and presence of species found at 200 sampling sites partitioned amongst 70 mesohabitat units in the San Saba River, TX during 2018 surveys.

Species	Abundance (n)	Presence (# Sites)	Presence (# Mesohabitat units)
***Lampsilis bracteata***	327	70	24
***Utterbackia imbecillis***	299	67	25
***Cyrtonaias tampicoensis***	104	12	3
***Amblema plicata***	48	4	4
***Tritogonia verrucosa***	37	7	7
***Quadrula quadrula***	20	12	8
***Pustulosa petrina***	13	4	4
***Pustulosa pustulosa***	9	1	1
***Fusconaia iheringi***	2	1	1

### Data collection

Existing data from 200 sites sampled every 100 m in a 20 km segment of the upper San Saba River was used for this study (outlined above; Fig 1). All freshwater mussel data was collected under a scientific collection permit issued to Astrid Schwalb by the Texas Parks and Wildlife Department. Survey methods and data are publicly available in [60, 61] and in S1 Appendix.

### HEC-RAS model

**Fig 2:**
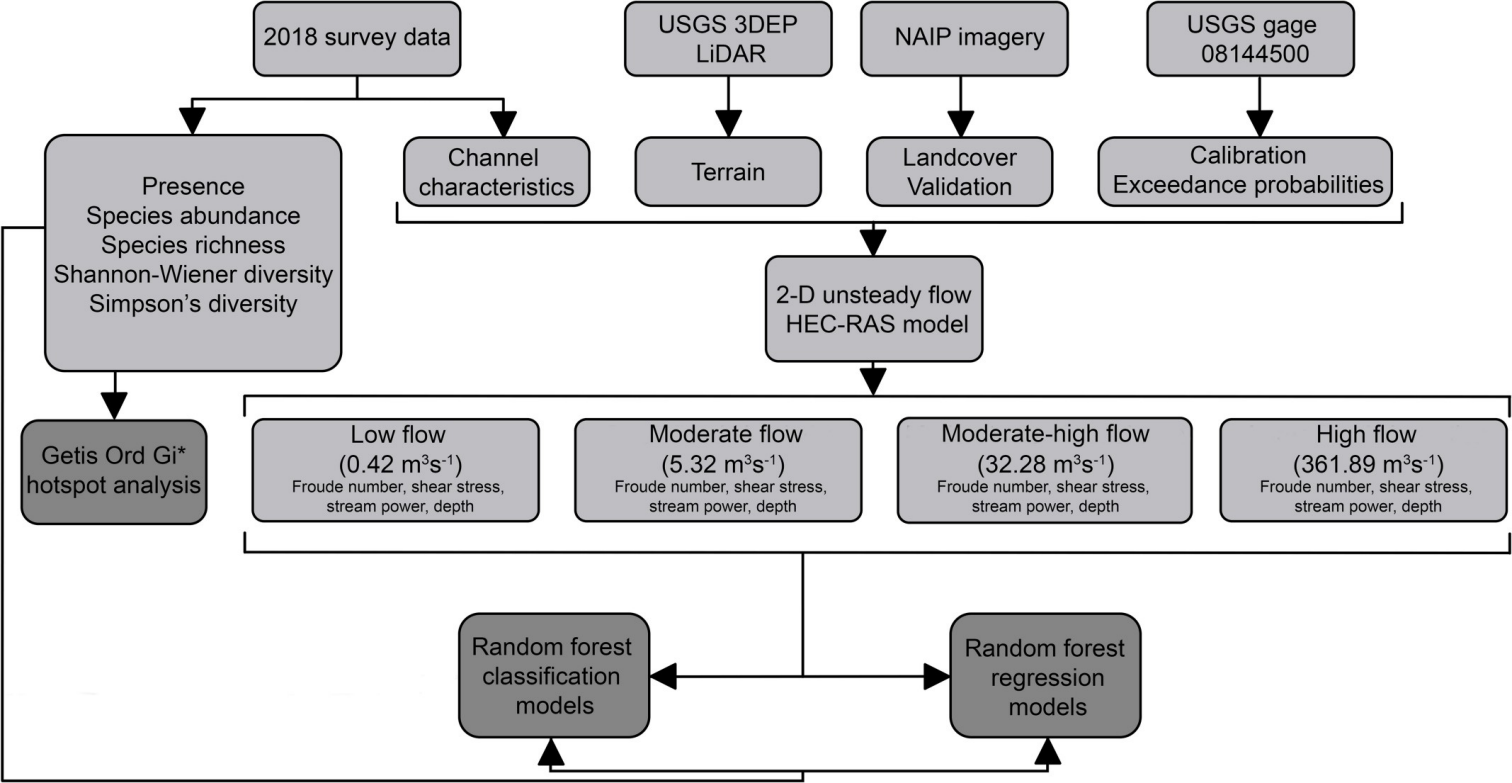
Data flow diagram describing inputs and outputs of data analysis, including the data used to create a two-dimensional HEC-RAS model to simulate flow conditions in a segment of the San Saba River, TX, USA. For additional information about construction of the model and data analysis, please see the methods section and S1 Appendix.

A two-dimensional (2-D) hydraulic model for the study segment was prepared using the Hydrologic Engineering Center’s River Analysis System (HEC-RAS) software (v. 6.2, Hydrologic Engineering Center, Davis, CA 95616–4687 USA). Discharge data recorded at United States Geological Survey (USGS) streamflow gage 08144500 near Menard, TX, located approximately 15 km downstream of the study segment, were used to inform model building (Fig 2) [58]. Light detection and ranging (LiDAR) data was collected by the USGS 3-D elevation program (3DEP) between February and April 2018 with 1 m resolution. These data were used to create a terrain in HEC-RAS (Fig 2) [62]. Flows during this period were comparable to flows during field data collection (0.45–0.71 m^3^s^-1^ versus 0.18–0.51 m^3^s^-1^, respectively). Landcover in the floodplain was identified using aerial imagery [63]. Details regarding how the model was created can be found in S1 Appendix and the model is publicly available for download via [64].

The model was calibrated to 1) match velocities and depths recorded during field measurements and inundation at the time of LiDAR collection and 2) match inundation extents from aerial photography at higher flows [63]. Post-calibration, the overall root mean squared error of the model was approximately 0.08 m s^-1^ for velocity and 0.17 m for depth. Error at individual cross sections was also evaluated on an individual basis for accuracy, and one site at the end of the segment was removed from subsequent analyses due to high error resulting from hydraulic model boundary conditions.

In addition, to assess how uncertainty in hydraulic conditions at flows higher than the flow during data collection (0.42 m^3^s^-1^) may have influenced the results, a sensitivity analysis was performed by re-running simulations using minimum and maximum Manning’s coefficients for selected land uses in the floodplain (S1 Table) [65]. This helped approximate how unrecognized differences in floodplain characteristics may have influenced hydraulic conditions during higher flows in the absence of discharge data or aerial imagery for flows that result in floodplain inundation.

### Simulated flow events

In the context of the upper San Saba River, we defined a low flow as any discharge below median daily flow (0.62 m^3^s^-1^), and a high flow as any discharge at least 5x greater than median daily flow. To estimate the magnitude of hydraulic variables during flow events, flows equivalent to the calibrated flow (0.42 m^3^s^-1^ hereafter low flow; ∼0.7x median daily flow), as well as flows with an exceedance probability of 10% and 50% were simulated using an unsteady flow simulation with the 2-D HEC-RAS model. Flows of this magnitude were considered because they represent conditions that occur on a relevant time scale for many long-lived mussels. In addition, all of these flows occurred in the recent past (*i*.*e*., 0–14 years prior to data collection, Table 3), so they can reasonably be expected to have influenced mussel populations during 2018 when data was collected. Exceedance probabilities were calculated using daily average streamflow data from 1916 to 2021 (available from USGS gage 08144500) [58] using the Hydrologic Engineering Center’s Ecosystems Function Model (v.5.0, Hydrologic Engineering Center, Davis, CA 95616–4687 USA). Due to relatively infrequent flows of these magnitudes occurring in the study area over the last two decades, the 50% exceedance probability for the study segment during 1998–2018 was also calculated and used in further analyses.

**Table 3:** Flows simulated using HEC-RAS, including exceedance probabilities for USGS gage 08144500 (San Saba at Menard) for both the period of record (1916–2022) and 1998–2018 and date and magnitude of last exceedance [58].

Name	Discharge (m^3^s^-1^)	Exceedance Probability (%)	Time period	Date of last exceedance	Peak daily streamflow recorded (m^3^s^-1^)
**Low flow**	0.42	NA	1916–2022	July 13th, 2018	0.43
**Moderate flow**	5.32	50	1998–2018	May 20th, 2016	6.88
**Moderate-high flow**	32.28	50	1916–2022	May 18th, 2016	45.31
**High flow**	361.89	10	1916–2022	November 17th, 2004	761.72

A flow of 0.42 m^3^s^-1^ was representative of discharge at the time of data collection (July 2018).

### Selected discharges

For USGS gage 08144500 (Menard, TX) during the period of record (1916–2022), the 50% exceedance probability of discharge was determined to be 32.28 m^3^s^-1^ (∼50x median daily flow; hereafter, moderate-high flow, Table 3) and the 10% exceedance probability was 361.89 m^3^s^-1^ (∼600x median daily flow; hereafter high flow, Table 3). For 1998–2018 (two decades preceding data collection), the 50% exceedance probability was 5.32 m^3^s^-1^ (∼10x daily flow; hereafter moderate flow, Table 3), which was last exceeded in May 2016, approximately two years prior to data collection. The high flow discharge was last exceeded in November 2004 (Table 3). All three of these discharges are considered high flows for the purposes of this study.

### Data analysis

The HEC-RAS model was used to compare hydraulic conditions during high and low flows to field observations of mussel distribution. Following the hydraulic simulation, the depth, shear stress, Froude number, and stream power results layers were exported to ArcGIS Pro, where all further geospatial analyses were conducted (v. 2.50, ESRI, Redlands, CA 92373–8100 USA; Table 4). Field sites were recorded as a single GPS point. To represent the field sampling search area for spatial analysis, the GPS point was transformed into a cross section or river transect with a five-meter buffer in the upstream and downstream direction. The mean, maximum, and median statistics for each hydraulic variable within estimated sampled areas for each cross section were calculated using the ‘Zonal Statistics as Table’ tool and exported for analysis.

**Table 4:** Complex hydraulic variables calculated in the U.S. Army Corps of Engineers Hydrologic Engineering Center’s River Analysis System (HEC-RAS).

Variable	Equation	Definition	Source
**Shear stress (N m** ^**-2**^ **)**	τ = γ*R*_*T*_*S*_*f*_	Force applied parallel to the riverbed, computed as specific weight of water (γ) times hydraulic radius (*R*_*T*_) times the energy grade line slope (*S*_*f*_).	[66, 67]
**Stream power (N-s m** ^**-2**^ **)**	*Ω* = *v*τ	Ability of a flow to do work, computed as cross-section average velocity (*v*) times cross-section shear stress (τ)	[18, 67]
**Froude number (dimensionless)**	$Fr=\frac{v}{\sqrt{gD}}$	Ratio of inertial to gravitational forces, computed as velocity (*v*) divided by the square root of the gravitational constant (*g*) times distance (*D*) between computational points	[66, 67]

Data analysis was conducted by site for all analyses and by site and mesohabitat unit (*e*.*g*., riffle, pool, run) for mussel presence to examine differences based on scale (Fig 1). Surveyed sites were grouped together based on the mesohabitat recorded during sampling in [60] and compared to aerial imagery [63]. If a site was identified as the same mesohabitat type as a site directly upstream or downstream, with a continuous channel form in aerial imagery, the sites were combined into a single channel unit based on that mesohabitat. Catch per unit effort (CPUE) and species per unit effort (SPUE) were calculated based on cumulative effort (in person hours (p-h)). Grouping sites resulted in search efforts between 0.5 p-h and 5.5 p-h, with an average search effort of 1.44 p-h. Subsequent analyses were carried out using R software (v.4.2.1, R Core Team, Vienna, Austria) or ArcGIS Pro software. Differential use of mesohabitat types was evaluated using chi-squared analysis and calculation of a preference index [68].


$$\text{Preference}\;\text{index}=\frac{\%\;\text{habitat}\;\text{occupied}}{\%\;\text{habitat}\;\text{available}}$$ (1)

Simpson’s and Shannon-Wiener diversity indices were calculated for each site using the ‘vegan’ package in R [69]. Multiple diversity metrics were used to gain a more robust understanding of diversity patterns [70]. Mussel richness is simply the number of species present, whereas diversity also includes a measure of the evenness or relative abundance of species at a site. The Simpson’s diversity index was computed with:

$$\left(1-\text{D}\right)=1-\text{log}\sum_{\text{i}-1}^{\text{m}}{\text{p}}_{\text{i}}^{2}$$ (2)

where *P*_*i*_ is equal to the relative abundance of species *i* and *m* is the total number of species [70]. Sites with no mussels were assigned a Simpson’s diversity of zero.

The Shannon-Wiener diversity index was computed with:

$$\left({\text{H}}^{\prime }\right)=-\sum_{i-1}^{m}p_{i\cdot \log\left(P_{i}\right)}$$ (3)

where *P*_*i*_ is equal to the relative abundance of species *i* and *m* is the total number of species [70].

Getis-Ord Gi* hotspot analysis with inverse distance conceptualization was conducted in ArcGIS Pro software to identify hotspots of species richness and diversity (Shannon-Wiener and Simpson’s) for individual sites [71]. Getis-Ord Gi* hotspot analysis works by comparing richness or diversity at sites with other sites throughout the segment to identify areas where high values are spatially clustered (*i*.*e*., hotspot) or spatially dispersed (*i*.*e*., coldspot) [72]. With inverse distance conceptualization, all sites are treated as neighbors, but sites that are closer to each other have more influence than sites that are farther away [73]. Ecologically, these hotspots represent areas that contain distinctive assemblages of biota that harbor significantly more species and higher diversity than the rest of the study area.

To reduce the number of statistical tests run on the data, hotspots of richness and diversity were pooled, and Wilcoxon tests were used to compare hydraulic variables at selected flows in hotspots versus other areas in the segment [74]. This allowed us to examine how hydraulic conditions differed at sites harboring significantly higher mussel diversity and richness. Wilcoxon tests are a useful alternative to parametric testing when assumptions like normality and homoscedasticity are not met and when working with unequal sample sizes [75], both of which applied to this dataset. In R Studio, the compare means function in the ‘ggpubr’ (v.0.40) package was used to carry out analyses [76]. The threshold for significance during hotspot analysis was 0.05.

Random forest (RF) modeling was used to examine the influence of hydraulic conditions on freshwater mussels within the study segment. RF serves as a robust analysis method akin to classification and regression trees, which does not assume normality, linearity, or homoscedasticity, is less sensitive to spatial autocorrelation, and can handle multicollinearity, issues that are often inherent in ecological data [76, 77]. RF can handle both classification and regression tasks and works by generating a series of low-correlated bootstrapped trees using randomly selected variables and averaging all trees to obtain estimates of error and variable importance [76]. For each tree in the forest, bootstrap aggregation (also known as bagging) is used to randomly select a subset of the data (67%) as training data while retaining the remaining data (33%) for model testing, resulting in the ability to estimate out-of-bag (OOB) error rates for the model.

The ‘randomForest’ package [78] in R Studio was used to investigate the influence of hydraulic conditions at multiple flows (*i*.*e*., low, moderate, moderate-high, and high) on freshwater mussel populations. The number of variables tested in each tree (*mtry* in ‘randomForest’) and the terminal node size of trees were tuned using the ‘tuneRanger’ and ‘ranger’ packages (also required ‘mlr’ and ‘caret’ packages) [79–83], and ten thousand trees were used for each model to ensure model convergence [81]. Optimal *mtry* was allowed to vary between one and three. For each selected flow, RF classification was used to understand the influence of hydraulic conditions on mussel presence, and RF regression was used to examine influences on log(x+1) abundances (catch per unit effort or CPUE) of species for which greater than one hundred individuals were found during surveys. Log(x+1) abundance was used to reduce dispersion in the data [83]. Each analysis was run ten times using different random seeds, and final estimates were obtained by averaging the results of each run.

By default, the relative importance of variables in RF models is determined using estimates of the mean decrease in model accuracy if a variable was dropped from the analysis (classification) or the increase in mean standard error (MSE) if the values of a variable were randomly reordered in out-of-bag samples (regression) [84]. However, because variable importance scores can be influenced by highly correlated predictors (which were present in this dataset), the conditional permutation importance (CPI) of each predictor variable was calculated using the ‘permimp’ package in R [85]. The CPI is a measure of the influence that a given predictor has on a response in addition to other predictors in the model [85], and operates in the same manner as the initial permutation importance calculated by the random forest, but partitions the predictor space based on additional predictors that may be interacting with the main predictor, such that importance values of a given predictor take into account potential interactions with other predictors [85]. Values of CPI can measure the relative importance of variables in a model in relation to each other [85]. Variables with negative CPI values, indicating that the model performed better when the variable was randomly permuted, were removed and RF models were recalculated according to the reduced number of variables [85]. The default threshold of 0.95 was used for determining association of predictors [85].

## Results

### Segment hydraulic conditions and sensitivity analysis

Hydraulic conditions varied across sites and across modeled flows, with average overall hydraulic conditions that mussels would be exposed to in the segment increasing in magnitude as discharge increased (S2 Table and S1–S4 Figs). The sensitivity analysis indicated that differences in estimated hydraulic conditions given changes in roughness values were relatively minimal at all flows (S3 Table). The largest differences occurred for shear stress during the high flow (S3 Table). Shear stresses of the magnitude recorded for this flow were approximately equivalent to the critical shear stress (given uniform substrate) that would initiate movement of small cobbles (median grain sizes between 64 and 128 mm), and the uncertainty in shear stress estimates could potentially equate to differences in movement of objects ranging in size from very coarse gravel to large cobble (median grain sizes between 32 and 256 mm) [86]. Despite this, the relative relationships of conditions at simulated flows were maintained across roughness values, with higher values for all variables increasing as flows increased.

### Mesohabitat preferences and hotspot analysis

**Fig 3:**
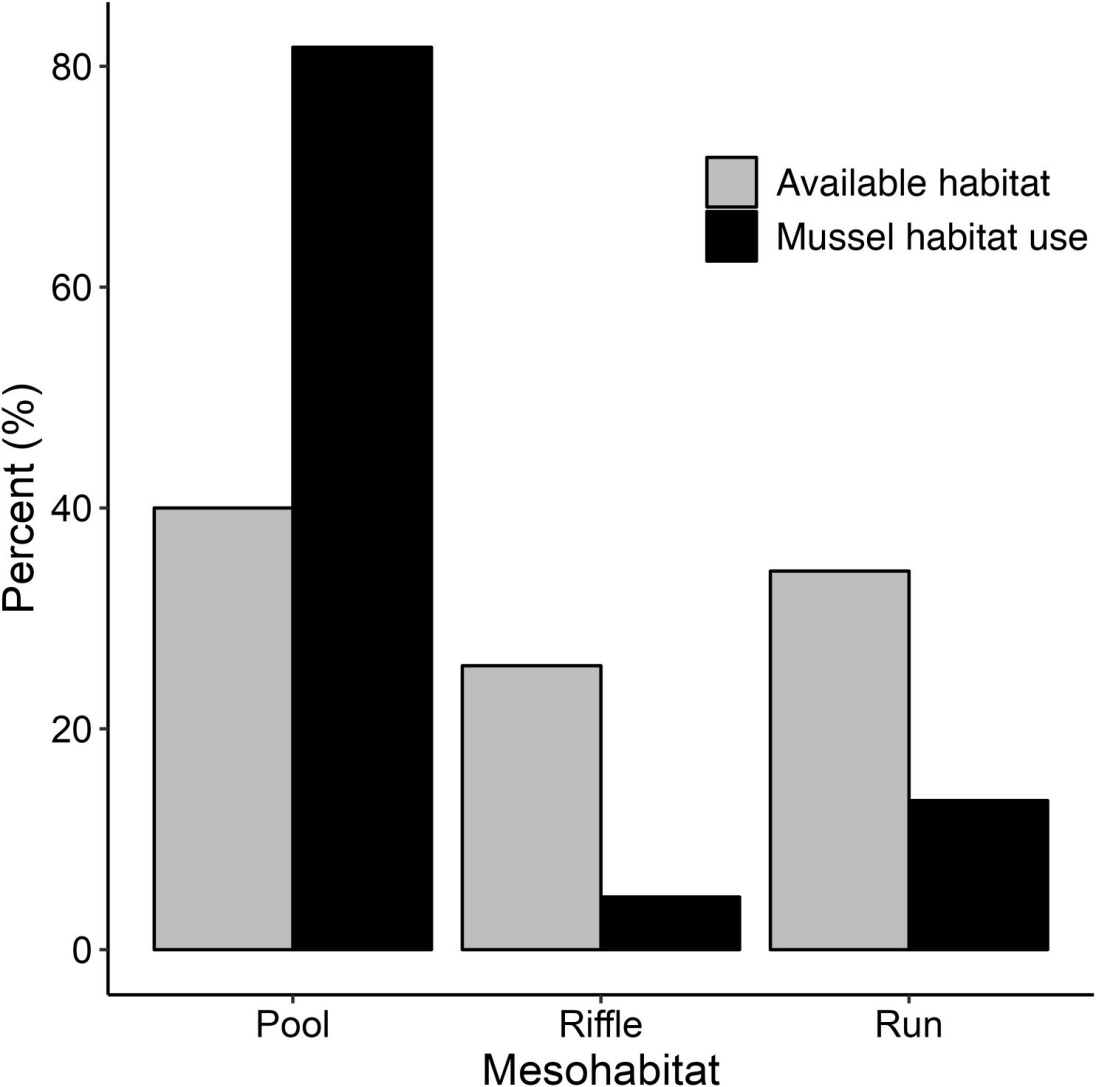
Habitat preferences of freshwater mussels [67], as shown by percent of available habitat sampled (*i*.*e*., pool, riffle, run) compared to percent of the total mussel abundance found in each mesohabitat type. The study segment was made up of 28 unique pools, 24 runs, and 18 riffles.

Our field data revealed that mussels in the study segment exhibited distinct mesohabitat preferences given that observed mussel abundances were not split proportionally amongst available mesohabitats (28 pools, 24 runs, and 18 riffles in the study segment; χ2 = 628.5, df = 2, p < 0.001). The preference index indicated that mussels preferentially occupied pool habitats, with nearly 82% of mussels found in pool habitats, despite pools making up only 40% of available surveyed habitat (Fig 3). In contrast, approximately 13.5% of mussels were found in runs and 4.5% in riffles, despite each habitat making up 34% and 26% of available habitat, respectively.

**Fig 4:**
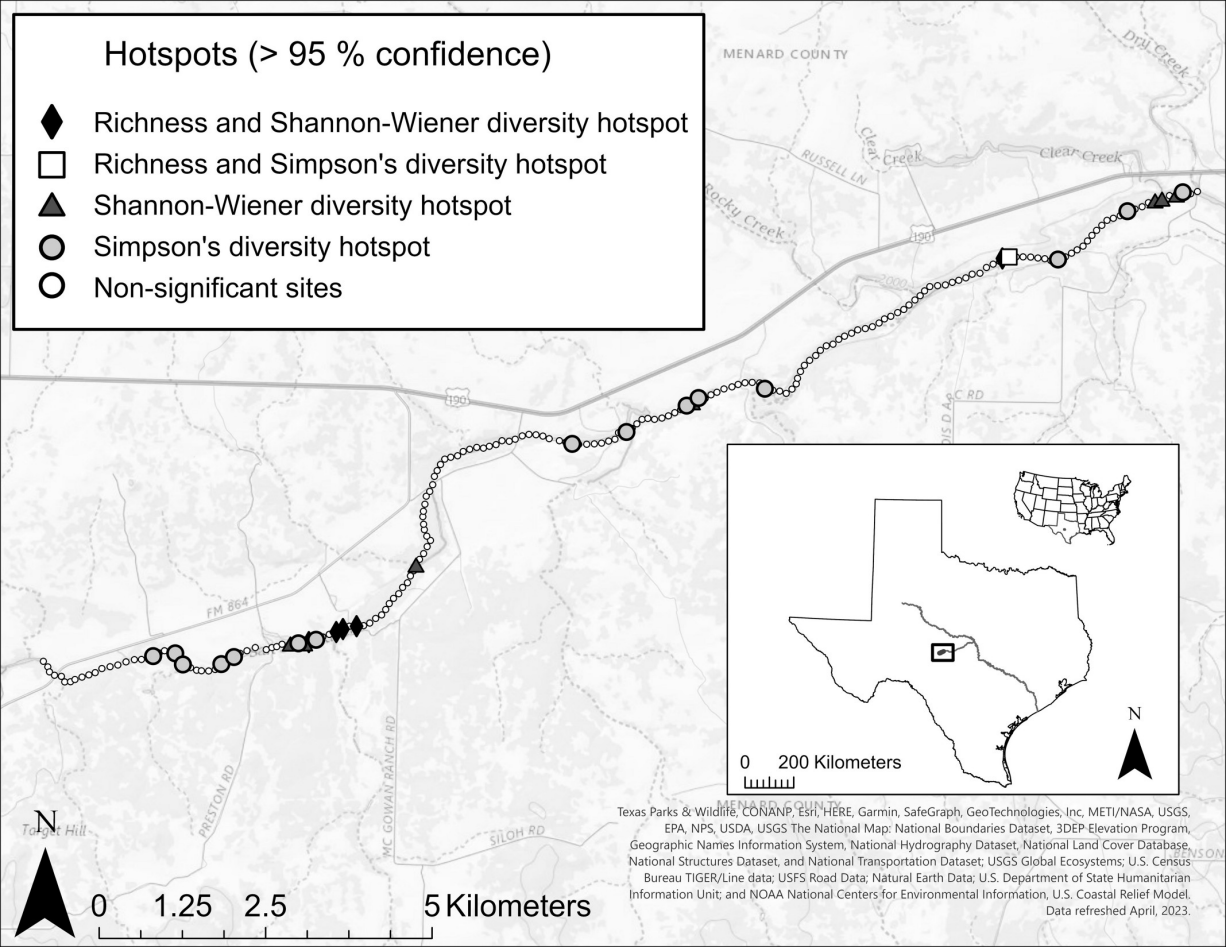
Getis Ord Gi* hotspots of species richness, Shannon-Wiener diversity, and Simpson’s diversity within the study segment in the San Saba River, TX. Hotspots represent spatially clustered areas of high richness and diversity compared to other sites within the study segment. In the upper San Saba River, hotspots occur in flow refuges where stable conditions may promote and protect diverse or species-rich communities. Map services and data available from U.S. Geological Survey, National Geospatial Program.

**Fig 5:**
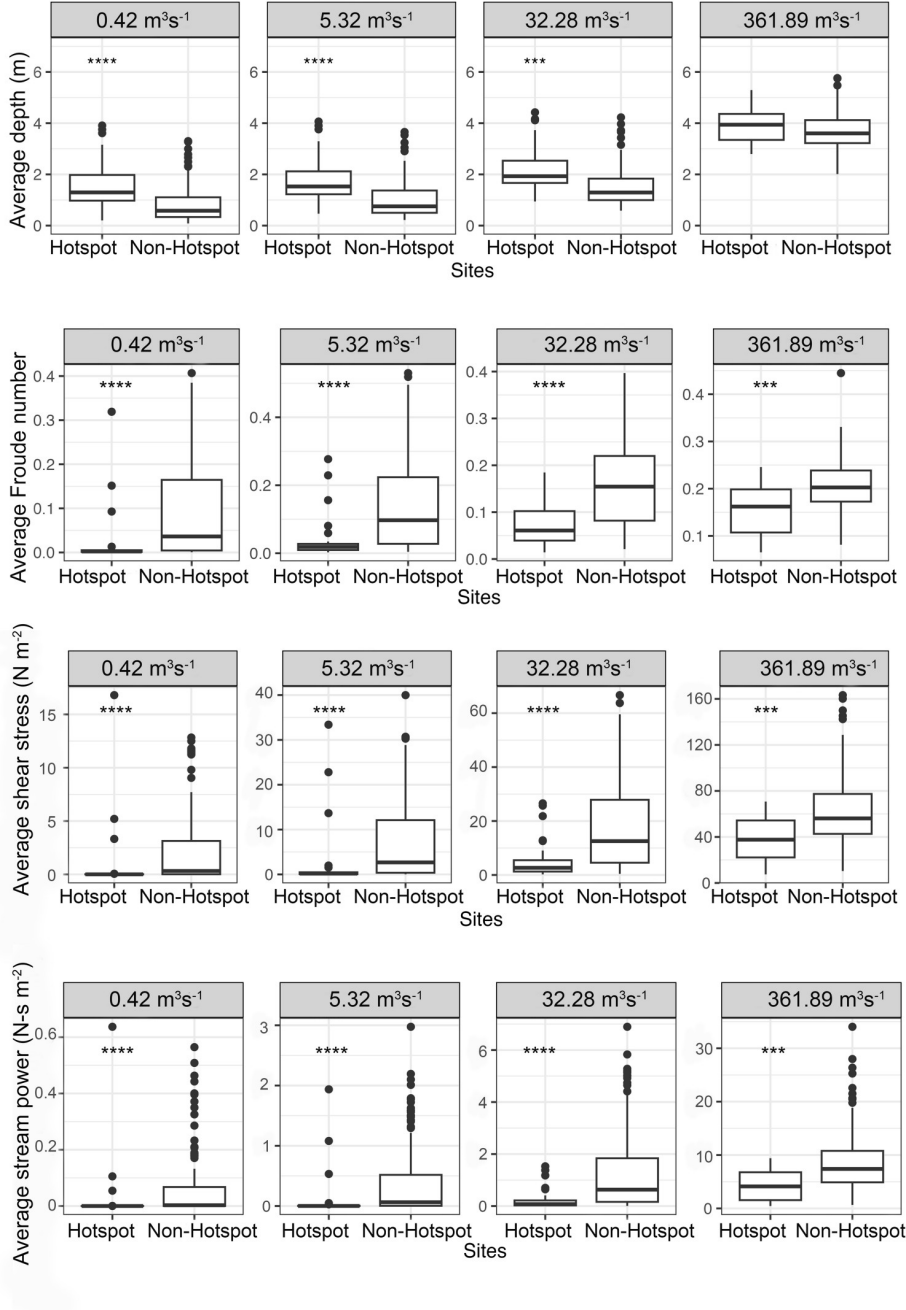
Results of Wilcoxon tests comparing average hydraulic conditions during low (0.42 m^3^s^-1^), moderate (5.32 m^3^s^-1^), moderate-high, (32.28 m^3^s^-1^), and high (361.89 m^3^s^-1^) flow conditions in a study segment in the San Saba River, TX at hotspots of richness and diversity (Shannon-Wiener and Simpson’s) versus non-hotspot sites in the study segment. Hotspots represent sites that hosted significantly (>95% confidence) higher species diversity or richness compared to neighboring sites within the segment. In the upper San Saba River, stable conditions may promote and protect diverse and species-rich communities. Asterisks indicate significant differences.

Across the 200 sampled sites, 28 hotspots (>95% confidence) of richness (n = 6) and Shannon-Wiener (n = 12) or Simpson’s (n = 16) diversity were identified within the study segment (Fig 4). Average depth at hotspots was significantly higher than non-hotspot areas at all flows except the highest flow (p<0.001 for low, moderate, and moderate-high flows, p = 0.06 for high flow), while average Froude number, shear stress, and stream power were significantly lower at all flows at hotspots versus non-hotspots (p<0.01 for all flows and all variables; Fig 5).

### Random forest models

In general, response variables (mussel presence and log(x+1) species abundances) were all positively related to depth and negatively related to Froude number, shear stress, and stream power at all flow magnitudes and at both the site and mesohabitat scale (S4 and S5 Tables). A select number of measurements did not follow this pattern, but in all instances that this occurred, correlations were low (-0.07 < r < 0.19) and non-significant (S4 and S5 Tables).

**Fig 6:**
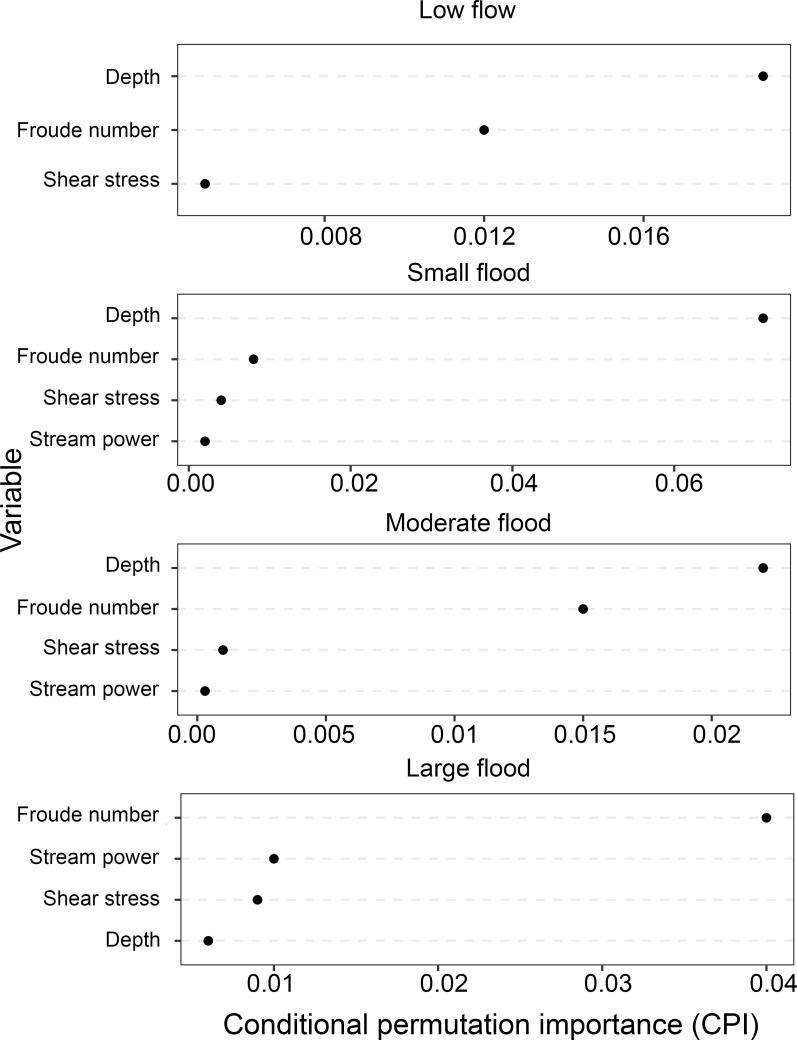
Variable importance plots for random forest classification examining the response of freshwater mussel presence to modeled hydraulic variables during a low (0.42 m3s-1), moderate (5.32 m3s-1), moderate-high (32.28 m3s-1), and high flow (361.89 m3s-1) in a 20 km segment of the San Saba River, TX, USA. Variable importance was determined using conditional permutation importance (CPI) because some predictor variables were found to be highly correlated [81]. Note: CPI should not be compared across different RF models.

Overall, the selected hydraulic conditions produced RF classification models with relatively good accuracy at all flows for both site and mesohabitat scales (Table 5). Random forest models were able to successfully classify sites with mussel presence versus absence between 67 and 79% of the time at the site scale and 60 to 70% of the time at the mesohabitat scale (Table 5). The highest accuracy rate for sites and mesohabitats was associated with the moderate flow scenario, followed by the low flow, the moderate-high flow, and the high flow (Table 5). Depth and Froude number were the most important variables in RF models for all flow scenarios except the high flow, for which Froude number and stream power were most important (Table 5 and Fig 6). Stream power and shear stress at the mesohabitat level and stream power at the site level were removed from the low flow model due to negative CPI (Table 5). In addition, depth was removed from the high flow model at the mesohabitat level (Table 5).

**Table 5:** Results of random forest (RF) classification models examining the response of freshwater mussel presence to hydraulic conditions at simulated flows using site-level and mesohabitat-level data.

		Classification results						
Scale	Flow	Discharge (m^3^s^-1^)	Accuracy rate (%)	OOB error rate (%)	False positive rate (%)	False negative rate (%)	mtry	Minimum node size
**Site results** **(n = 200)**	Low	0.42	77.3	22.7	24.2	21.3	1	27
	Moderate*	5.32	78.6	21.4	25.9	17.31	2	40
	Moderate-high	32.28	72.6	27.4	27.4	30.29	3	29
	High	361.89	67.7	32.3	36.3	28.56	1	40
**Mesohabitat results** **(n = 70)**	Low	0.42	68.6	31.4	28.6	34.3	1	13
	Moderate*	5.32	70	30	22.9	37.1	1	14
	Moderate-high	32.28	67.1	32.9	28.6	37.1	2	14
	High	361.89	60.1	39.9	34.3	34.9	1	2

*Most accurate model. Each model contained 10000 trees and was replicated 10 times using different random seeds to obtain average error rates and importance. Mtry is the number of randomly selected variables to be included in each tree, and out-of-bag (OOB) error is the average accuracy of the model built using training data (67% of data) when applied to test data (33% of data).

Regression tree models were created for the three species with abundances greater than 100 individuals, *L*. *bracteata*, *U*. *imbecillis*, and *C*. *tampicoensis*. For these species at the site scale, hydraulic variables at varying flows were able to explain between 0.6 and 55% of the variation in log(x+1) CPUE at sites (Table 6).

**Table 6:** Results of random forest (RF) regression models examining the response of freshwater mussel log(x+1) species’ abundances to hydraulic conditions at simulated flows.

	Regression							CPI		
Model	Flow	Discharge (m^3^s^-1^)	R2	MSE	mtry	Minimum node size	Depth	Froude	Shear stress	Stream power
***Lampsilis bracteata* abundance**	Low	0.42	4.8	1.0	3	2	0.074	0.003	0.005	0.002
	Moderate*	5.32	13.8	0.9	3	2	0.086	0.036	0.027	NA
	Moderate-high	32.28	9.1	1.0	1	40	0.02	0.054	NA	0.013
	High	361.89	0.6	1.1	1	40	0.001	0.08	NA	0.02
***Utterbackia imbecillis* abundance**	Low	0.42	24.1	0.8	1	29	0.009	0.031	0.022	0.019
	Moderate*	5.32	27.2	0.8	1	40	0.038	0.045	0.007	0.007
	Moderate-high	32.28	18.4	0.9	1	29	NA	0.585	NA	NA
	High	361.89	12.7	0.9	2	37	NA	0.060	0.045	0.012
***Cyrtonaias tampicoensis* abundance**	Low	0.42	44.7	0.2	3	19	NA	0.005	0.157	0.159
	Moderate*	5.32	54.5	0.2	3	2	0.041	0.012	0.06	0.055
	Moderate-high	32.28	51.4	0.2	2	2	0.032	0.025	0.052	0.049
	High	361.89	52.1	0.2	2	27	NA	0.007	0.07	0.20

*Best model. Results represent mean squared error rates, explanation rates (R^2^), and variable importance (CPI) across all trees in the random forest. The conditional permutation importance (CPI) represents the relative importance of a predictor in the calculated RF model given the influence of other predictors. Variables with negative CPI values, indicating that the model performed better when the variable was randomly permuted, were removed and RF models were recalculated according to the reduced number of variables. Note: CPI should not be compared across different RF models.

RF models for *C*. *tampicoensis* were most successful, and over 50% of variation in log transformed CPUE could be explained by hydraulic variables at each of the three high flow conditions. For *C*. *tampicoensis*, shear stress and stream power were the most important variables at all flows (Table 6 and S5 Fig). Hydraulic variables were able to explain between 12 and 27% of variation in log transformed *U*. *imbecillis* CPUE, with the highest amount of variation explained for moderate and low flows (Table 6). Froude number was the most important variable for *U*. *imbecillis* abundance at all flows (Table 6 and S5 Fig). The smallest amount of variation could be accounted for in RF models for *L*. *bracteata* (∼0.6–14%; Table 6). Depth and Froude number were the most important variables for *L*. *bracteata* during moderate and moderate-high flows, whereas depth and shear stress were more important at low flows and Froude number and stream power were more important during high flows (Table 6 and S5 Fig).

## Discussion

This study provides valuable insight into how the distribution of sedentary freshwater species may be influenced by extreme conditions and rare events. In this case, we examined hydraulic conditions during both high and low flows, including conditions when hydraulic measurements would be impossible or extremely difficult to obtain given in-stream conditions. This is the first study that combined a hydraulic model with survey data on the distribution of mussels from a 20 km segment sampled relatively continuously, and one of few studies that have examined the influence of hydraulic conditions in the context of bedrock-dominated systems (see [15]).

### Hotspots of richness and diversity

Regardless of flow magnitude, hotspots of richness and diversity tended to occur in deeper areas that were sheltered from unfavorable flow conditions (*i*.*e*., high shear stress, stream power, and Froude number), which likely helps protect mussels from dislodgement during high flows [11, 15, 39] and may also serve as an important refuge from high temperatures and low dissolved oxygen at lower flows [15, 87, 88]. It is well known that hydraulic conditions affect richness and diversity of riverine organisms [89, 90]. However, the use of deeper areas like pool habitats as refuge during both low and high flows may be relatively unique to bedrock-dominated systems, as pools in finer sediment systems often experience high scour during high flow events that may prevent successful long-term mussel colonization [15, 91].

### Factors influencing mussel presence and distribution

Mussel presence across the study segment was predicted relatively accurately based solely on hydraulic conditions, which is consistent with other studies that have been successful in predicting mussel presence with complex hydraulic variables. Model accuracies as high as 71–76% [28] and 70–91% accuracy [16] have been reported in previous literature. Moderate and low flow models had the highest accuracy in this study, which indicates that conditions that occur more frequently are an important control for mussel distribution in streams, and especially in segments where flow intermittency may occur. Indeed, National Agricultural Inventory Program aerial imagery for the study area indicated that several sites may have partially or completely dried in recent years, which may be why hydraulic conditions during these more common flows had such a high predictive power [63, 87].

Studies in some other alluvial dominated systems have suggested that complex hydraulic variables during low flows are less important for mussels compared to higher flows [17]. In bedrock dominated systems, however, pools may serve as refuges for mussels during both low and high flows because the stability of the substrate prevents issues like scour that may limit pool occupancy in alluvial dominated streams [15]. In addition, extensive use of flow refuges like bedrock crevices or sedge mats may allow mussel species such as *Margaritifer*a *falcata* to occupy pools despite extreme flow conditions [15]. Use of flow refuges like bedrock crevices and vegetation is commonly observed in pools in the San Saba River and other bedrock-dominated streams in central Texas, especially for the two most abundant species in this study (*L*. *bracteata* and *U*. *imbecillis* [60]). Hence, if many of the mussels found during this study occupied flow refuges in pools that protect them from the effects of shear stress and stream power during high flows, conditions during low flows such as adequate depth, which influences refuge potential during drought, may become more influential [87, 88]. This could explain why models based on the lowest flows in this study were most accurate despite the adverse effects high flows may have, and why depth was frequently ranked as one of the most important variables despite its mixed success in other systems [17, 44].

In addition to depth, Froude number was consistently selected as one the most important variables across several RF models, which was also found for freshwater mussels in the Amazon Basin [19]. While shear stress is frequently cited as one of the most influential hydraulic variables limiting mussel distribution [17, 26, 28], Froude number may be a better predictor of mussel distribution than shear stress in some rivers. Froude number plays an influential role in both bedload movement and turbulence, which similarly to shear stress, may influence habitat stability and suitability for mussels [21, 22].

RF models had stronger predictive power with site data compared to mesohabitat data. If mussel distribution across sites was driven largely by randomness, then we would expect higher predictive power with a lower sampling size (*i*.*e*., mesohabitat data). However, the opposite was the case, which suggests that smaller-scale patterns in hydraulic conditions that more directly affect mussels are important driving factors of their patchy distribution.

Despite the higher accuracy of low and moderate flow models, the moderate-high and high flow models also performed well. The RF model for the high flow was able to successfully classify nearly 67% of sites despite flows of this magnitude having last occurred more than a decade prior. The success of this model despite the temporal gap in flows suggests that hydraulic conditions during high flow events may have long-term impacts on mussel populations that persist even after sufficient time for some recolonization has occurred. The ability of floods and high flow events to induce mortality in freshwater mussels has been documented [12, 26], and the lasting impacts for mussel populations may have important implications for populations, even in areas where extreme flow events are rare.

### Individual species abundances

At the site scale, more than half of the variation in *C*. *tampicoensis* abundance could be explained by hydraulic conditions during moderate, moderate-high, and high flows. The most important variables influencing *C*. *tampicoensis* abundance at all flows tended to be stream power and shear stress. This species is considered a more lentic species and is often found near banks and in backwater areas [92], and its distribution was rather limited in the study area (only found at 12 sites). In contrast, *U*. *imbecillis*, another lentic species, was more abundant and widespread throughout the segment. The use of flow refuges, which provide shelter from high shear stress and bed mobility during floods and high flow events [12, 15] likely explains why RF models were less successful in explaining variation in the abundance of both *U*. *imbecillis* and *L*. *bracteata*, which are often observed occupying refuge habitats such as bedrock crevices (K. Cushway, personal observation).

### Limitations

Our study was limited by a lack of empirical data for high flows and groundwater inputs and diversions, and coarse lateral measurements. Despite these factors likely contributing to the error of our models, our predictive power was still relatively high, indicating that we were able to capture important ecological patterns and insights even with limited available data. Our analysis also represents a discrete range of flow conditions, whereas responses of freshwater mussel assemblages may actually vary more along a continuous gradient of flow conditions. Leveraging machine learning techniques to incorporate large amounts of continuous flow data may be a promising avenue of future research to further elucidate how freshwater mussels are influenced by variation in flow conditions.

## Conclusions

This study supports the importance of flow refuges for mussel persistence during both high and low flows [11]. The results suggest that flow refuges at different scales may be an important driver of mussel communities and potentially other sedentary riverine organisms (*e*.*g*., aquatic insects that build shelters or mostly sedentary fish). In bedrock-dominated systems, pools may provide refuge against drying [87] while also providing more stable substrate during high flows compared to alluvial systems [93]. In addition, smaller scale, species-specific flow refuges such as bedrock crevices or vegetation may help certain species persist in habitats that would otherwise be exposed to unfavorable hydraulic conditions. Differences in the hydraulic habitat needs of specific species highlight the need to understand how individual species may respond to flow events when managing species that may be threatened or endangered. Understanding the effects of high and low flows will be an important aspect of management and conservation for determining habitat suitability of organisms that are already imperiled and facing challenges associated with climate change, degraded habitats and altered flows. Multidisciplinary collaborations between different groups of researchers like ecologists and engineers can provide essential tools and outlooks like the model used in this study that may be invaluable for understanding these habitat needs, especially in the face of changing climates.

## Supporting information

S1 Table Selected, minimum, and maximum Manning’s coefficients [64] tested during sensitivity analysis for landcover present in the floodplain near the San Saba River, TX, U.S.A used in a 2D HEC-RAS model, publicly available at [64]. A sensitivity analysis was performed for simulated flows of 5.32 m^3^s^-1^, 32.28 m^3^s^-1^, and 361.89 m^3^s^-1^ in attempt to quantify uncertainty in hydraulic variables given unrecognized differences in land cover within the floodplain. Roughness values were adjusted in the floodplain only, while channel roughness values were maintained for all analyses. Table prepared by Aubrey Harris and Samantha Wiest.
(DOCX)

S2 Table Average ± standard deviation of hydraulic conditions for simulated flow events at 200 sampling sites in the upper San Saba River, TX. (DOCX)

S3 Table Estimates of median average hydraulic variables (median ± standard deviation) for 200 sites in the San Saba River, TX given minimum, selected, and maximum Manning’s coefficients for identified land uses in the floodplain of the study segment at simulated flows higher than the calibrated flow. Differences presented here represent potential differences in hydraulic conditions due to unquantified differences in flow behavior given floodplain inundation. Manning’s coefficients were chosen based on values suggested by [64].
(DOCX)

S4 Table Spearman correlation coefficients (r) between hydraulic variables and mussel indicators at the site scale. Indicators include mussel presence, log(x+1) species’ CPUE, SPUE, Shannon-Wiener diversity, and Simpson’s diversity. Correlations in bold print were significant after Bonferroni adjustment. The adjusted threshold of significance was p < 0.0002.
(DOCX)

S5 Table Spearman correlation coefficients (r) between hydraulic variables and mussel indicators at the mesohabitat scale. Indicators include mussel presence, log(x+1) species’ CPUE, SPUE, Shannon-Wiener diversity, and Simpson’s diversity. Correlations in bold print were significant after Bonferroni adjustment. The adjusted threshold of significance was p < 0.0002.
(DOCX)

S1 Fig Simulated depths (m) for an A) low (0.42 m^3^s^-1^), B) moderate (5.32 m^3^s^-1^), C) moderate-high (32.28 m^3^s^-1^), and D) high (361.89 m^3^s^-1^) flows in a study segment in the San Saba River, TX. Discharges were modeled in the Hydrologic Engineering Center’s River Analysis System (HEC-RAS) using a two-dimensional unsteady flow model built using survey data collected in 2018.
(ZIP)

S2 Fig Simulated Froude number (unitless) for an A) low (0.42 m^3^s^-1^), B) moderate (5.32 m^3^s^-1^), C) moderate-high (32.28 m^3^s^-1^), and D) high (361.89 m^3^s^-1^) flow in a study segment in the San Saba River, TX. The black line in each color scale represents critical flow (Froude = 1). Above the black line, flow is super-critical and below the line, flow is sub-critical. Discharges were modeled in the Hydrologic Engineering Center’s River Analysis System (HEC-RAS) using a two-dimensional unsteady flow model built using survey data collected in 2018. [64].
(ZIP)

S3 Fig Simulated shear stress (N m^-2^) for an A) low (0.42 m^3^s^-1^), B) moderate (5.32 m^3^s^-1^), C) moderate-high (32.28 m^3^s^-1^), and D) high (361.89 m^3^s^-1^) flow in a study segment in the San Saba River, TX. Discharges were modeled in the Hydrologic Engineering Center’s River Analysis System (HEC-RAS) using a two-dimensional unsteady flow model built using survey data collected in 2018 [64].
(ZIP)

S4 Fig Simulated stream power (N-s m^-2^) for an A) low (0.42 m^3^s^-1^), B) moderate (5.32 m^3^s^-1^), C) moderate-high (32.28 m^3^s^-1^), and D) high (361.89 m^3^s^-1^) flow in a study segment in the San Saba River, TX. Discharges were modeled in the Hydrologic Engineering Center’s River Analysis System (HEC-RAS) using a two-dimensional unsteady flow model built using survey data collected in 2018 [64].
(ZIP)

S5 Fig Variable importance plots for random forest regressions predicting abundances of *Cyrtonaias tampicoensis*, *Lampsilis bracteata*, and *Utterbackia imbecillis* using modeled hydraulic variables during a low (0.42 m^3^s^-1^), moderate (5.32 m^3^s^-1^), moderate-high (32.28 m^3^s^-1^), and high (361.89 m^3^s^-1^) flow in a 20 km segment of the San Saba River, TX, USA. Variable importance was determined using conditional permutation importance (CPI) because some predictor variables were found to be highly correlated [85]. Note: CPI should not be compared across different RF model.
(TIF)

S1 Appendix Additional methodology for field data collection and HEC-RAS model preparation. (DOCX)
