# Supplementary material for: Go with the flow: Impacts of high and low flow conditions on freshwater mussel assemblages and distribution
Source: PLoS One. 2024 Feb 15;19(2):e0296861. doi: 10.1371/journal.pone.0296861 (PMC10868800; doi:10.1371/journal.pone.0296861)
Supplement: S1 Table — A sensitivity analysis was performed for simulated flows of 5.32 m3s-1, 32.28 m3s-1, and 361.89 m3s-1 in attempt to quantify uncertainty in hydraulic variables given unrecognized differences in land cover within the floodplain. Roughness values were adjusted in the floodplain only, while channel roughness values were maintained for all analyses. Table prepared by Aubrey Harris and Samantha Wiest. (DOCX) [file pone.0296861.s001.docx]

**Table S1**. **Selected, minimum, and maximum Manning’s coefficients** [64] tested during sensitivity analysis for landcover present in the floodplain near the San Saba River, TX, U.S.A used in a 2D HEC-RAS model, publicly available at [64]. A sensitivity analysis was performed for simulated flows of 5.32 m^3^s^-1^, 32.28 m^3^s^-1^, and 361.89 m^3^s^-1^ in attempt to quantify uncertainty in hydraulic variables given unrecognized differences in land cover within the floodplain. Roughness values were adjusted in the floodplain only, while channel roughness values were maintained for all analyses. Table prepared by Aubrey Harris and Samantha Wiest.

| **Landcover Description** | **Chow 1959 description, which has minimum/normal/maximum ranges (**[**Manning's coefficients (orst.edu)**](http://www.fsl.orst.edu/geowater/FX3/help/8_Hydraulic_Reference/Mannings_n_Tables.htm)**)** | **Selected roughness** | **Minimum roughness** | **Maximum roughness** |
| --- | --- | --- | --- | --- |
| **Channel** | Main Channel or Mountain Streams |  |  |  |
| **Channel** | sluggish reaches, weedy, deep pools (normal) | 0.07 | 0.07 | 0.07 |
| **Channel2** | clean, winding, some pools and shoals, some weeds and more stones (maximum) | 0.05 | 0.05 | 0.05 |
| **Cobbly** | no vegetation in channel, banks usually steep, trees and brush along banks submerged at high stages no vegetation in channel, banks usually steep, trees and brush along banks submerged at high stages (maximum) | 0.07 | 0.07 | 0.07 |
| **Cobbly2** | sluggish reaches, weedy, deep pools (maximum) | 0.08 | 0.08 | 0.08 |
| **Ineffective Sec** | sluggish reaches, weedy, deep pools (normal) | 0.07 | 0.07 | 0.07 |
| **Ineffective Sec2** | sluggish reaches, weedy, deep pools (maximum) | 0.08 | 0.08 | 0.08 |
| **Ineffective Sec3** | very weedy reaches, deep pools, or floodways   with heavy stand of timber and underbrush (normal) | 0.1 | 0.1 | 0.1 |
| **Weedy Reach** | very weedy reaches, deep pools, or floodways   with heavy stand of timber and underbrush (between normal and maximum) | 0.12 | 0.12 | 0.12 |
| **Intermediate Zone** | Floodplains |  |  |  |
| **Grassy Floodway** | Light brush and trees in summer (minimum) | 0.07 | 0.04 | 0.08 |
| **Grassy Floodway2** | Medium to dense brush, in summer (minimum) | 0.1 | 0.07 | 0.16 |
| **Floodplain** | (Floodplains) |  |  |  |
| **Dense Woody** | Heavy stand of timber, down trees, little undergrowth (minimum) | 0.1 | 0.08 | 0.2 |
| **Sparse Shrub** | Light brush and trees, in summer (minimum) | 0.08 | 0.04 | 0.08 |
| **NoData** | Scattered brush, heavy weeds (minimum) | 0.06 | 0.035 | 0.07 |
