# Supplementary material for: Go with the flow: Impacts of high and low flow conditions on freshwater mussel assemblages and distribution
Source: PLoS One. 2024 Feb 15;19(2):e0296861. doi: 10.1371/journal.pone.0296861 (PMC10868800; doi:10.1371/journal.pone.0296861)
Supplement: S2 Table — (DOCX) [file pone.0296861.s002.docx]

**Table S2.** **Average ± standard deviation of hydraulic conditions for simulated flow events** at 200 sampling sites in the upper San Saba River, TX.

| **Discharge (m^3^s^-1^)** | **Depth (m)** | **Shear stress (N m^-2^)** | **Stream power (N-s m^-2^)** | **Froude number** |
| --- | --- | --- | --- | --- |
| **0.42** | 0.92 ± 0.77 | 1.90 ± 3.14 | 0.05 ± 0.10 | 0.08 ± 0.1 |
| **5.32** | 1.11 ± 0.78 | 6.54 ± 8.91 | 0.33 ± 0.55 | 0.12 ± 0.12 |
| **32.28** | 1.59 ± 0.76 | 16.03 ± 15.47 | 1.13 ± 1.40 | 0.15 ± 0.09 |
| **168.49** | 3.73 ± 0.74 | 58.16 ± 28.36 | 7.86 ± 5.27 | 0.2 ± 0.06 |
