# Supplementary material for: Go with the flow: Impacts of high and low flow conditions on freshwater mussel assemblages and distribution
Source: PLoS One. 2024 Feb 15;19(2):e0296861. doi: 10.1371/journal.pone.0296861 (PMC10868800; doi:10.1371/journal.pone.0296861)
Supplement: S3 Table — Differences presented here represent potential differences in hydraulic conditions due to unquantified differences in flow behavior given floodplain inundation. Manning’s coefficients were chosen based on values suggested by [64]. (DOCX) [file pone.0296861.s003.docx]

**Table S3.** **Estimates of median average hydraulic variables** (median ± standard deviation) for 200 sites in the San Saba River, TX given minimum, selected, and maximum Manning’s coefficients for identified land uses in the floodplain of the study segment at simulated flows higher than the calibrated flow. Differences presented here represent potential differences in hydraulic conditions due to unquantified differences in flow behavior given floodplain inundation. Manning's coefficients were chosen based on values suggested by [64].

| **Discharge** | **Variable** | **Minimum Manning's coefficient** | **Selected Manning's coefficient** | **Maximum Manning's coefficient** |
| --- | --- | --- | --- | --- |
| **5.32 m^3^s^-1^** | Depth (m) | 0.84 ± 0.78 | 0.88 ± 0.78 | 0.91 ± 0.78 |
| **Moderate flow** | Froude number | 0.09 ± 0.15 | 0.08 ± 0.13 | 0.07 ± 0.11 |
|  | Shear stress (N m^-2^) | 1.74 ± 8.86 | 1.92 ± 8.91 | 2.16 ± 10.53 |
|  | Stream power (N-s m^-2^) | 0.04 ± 0.63 | 0.04 ± 0.55 | 0.04 ± 0.57 |
| **32.28 m^3^s^-1^** | Depth (m) | 1.26 ± 0.78 | 1.37 ± 0.76 | 1.46 ± 0.76 |
| **Moderate-high flow** | Froude number | 0.16 ± 0.12 | 0.14 ± 0.09 | 0.11 ± 0.08 |
|  | Shear stress (N m^-2^) | 10.06 ± 15.32 | 11.25 ± 15.32 | 11.97 ± 20.11 |
|  | Stream power (N-s m^-2^) | 0.57 ± 1.55 | 0.55 ± 1.40 | 0.57 ±1.53 |
| **361.89 m^3^s^-1^** | Depth (m) | 3.38 ± 0.72 | 3.66 ± 0.74 | 3.96 ± 0.79 |
| **High flow** | Froude number | 0.22 ± 0.08 | 0.20 ± 0.06 | 0.18 ± 0.06 |
|  | Shear stress (N m^-2^) | 48.84 ± 29.21 | 53.63 ± 28.25 | 66.08 ± 42.14 |
|  | Stream power (N-s m^-2^) | 6.33 ± 5.88 | 6.70 ± 5.27 | 7.14 ± 6.40 |
