# Supplementary material for: Go with the flow: Impacts of high and low flow conditions on freshwater mussel assemblages and distribution
Source: PLoS One. 2024 Feb 15;19(2):e0296861. doi: 10.1371/journal.pone.0296861 (PMC10868800; doi:10.1371/journal.pone.0296861)
Supplement: S4 Table — Indicators include mussel presence, log(x+1) species’ CPUE, SPUE, Shannon-Wiener diversity, and Simpson’s diversity. Correlations in bold print were significant after Bonferroni adjustment. The adjusted threshold of significance was p < 0.0002. (DOCX) [file pone.0296861.s004.docx]

**Table S4**. **Spearman correlation coefficients (r) between hydraulic variables and mussel indicators at the site scale**. Indicators include mussel presence, log(x+1) species’ CPUE, SPUE, Shannon-Wiener diversity, and Simpson’s diversity. Correlations in bold print were significant after Bonferroni adjustment. The adjusted threshold of significance was p < 0.0002.

| **Site scale** | **Discharge** | **Depth (m)** | **Froude number** | **Shear stress (N m^-2^)** | | **Stream power**  **(N-s m^-2^)** |
| --- | --- | --- | --- | --- | --- | --- |
| **Presence** | 0.42 | **0.46** | **-0.44** | **-0.28** | -0.21 | |
|  | 5.32 | **0.48** | **-0.47** | **-0.33** | **-0.29** | |
|  | 32.28 | **0.45** | **-0.5** | **-0.38** | **-0.36** | |
|  | 361.89 | 0.25 | **-0.4** | **-0.28** | **-0.28** | |
| ***L. bracteata* CPUE** | 0.42 | 0.16 | **-0.28** | -0.19 | -0.14 | |
|  | 5.32 | 0.17 | **-0.32** | -0.24 | -0.2 | |
|  | 32.28 | 0.16 | **-0.3** | **-0.27** | -0.25 | |
|  | 361.89 | -0.01 | -0.1 | -0.12 | -0.14 | |
| ***U. imbecillis* CPUE** | 0.42 | **0.5** | **-0.37** | **-0.29** | -0.24 | |
|  | 5.32 | **0.52** | **-0.42** | **-0.33** | **-0.38** | |
|  | 32.28 | **0.51** | **-0.5** | **-0.42** | **-0.38** | |
|  | 361.89 | **0.28** | **-0.41** | **-0.34** | **-0.31** | |
| ***C. tampicoensis* CPUE** | 0.42 | **0.46** | -0.16 | -0.12 | -0.1 | |
|  | 5.32 | **0.42** | **-0.28** | -0.12 | -0.17 | |
|  | 32.28 | **0.42** | **-0.43** | -0.2 | -0.17 | |
|  | 361.89 | 0.22 | **-0.43** | **-0.35** | **-0.3** | |
| **Richness (SPUE)** | 0.42 | **0.4** | **-0.31** | -0.17 | -0.13 | |
|  | 5.32 | **0.42** | **-0.37** | -0.21 | -0.19 | |
|  | 32.28 | **0.4** | **-0.44** | **-0.34** | **-0.33** | |
|  | 361.89 | 0.16 | **-0.43** | **-0.35** | **-0.35** | |
| **Shannon-Wiener Diversity** | 0.42 | **0.33** | -0.24 | -0.15 | -0.13 | |
|  | 5.32 | **0.34** | **-0.27** | -0.17 | -0.16 | |
|  | 32.28 | **0.33** | **-0.36** | **-0.29** | **-0.27** | |
|  | 361.89 | 0.09 | **-0.37** | **-0.35** | **-0.32** | |
| **Simpson's Diversity** | 0.42 | **0.35** | **-0.26** | -0.18 | -0.15 | |
|  | 5.32 | **0.35** | **-0.3** | -0.2 | -0.18 | |
|  | 32.28 | **0.35** | **-0.37** | **-0.3** | **-0.28** | |
|  | 361.89 | 0.1 | **-0.36** | **-0.35** | **-0.32** | |
