# Supplementary material for: Go with the flow: Impacts of high and low flow conditions on freshwater mussel assemblages and distribution
Source: PLoS One. 2024 Feb 15;19(2):e0296861. doi: 10.1371/journal.pone.0296861 (PMC10868800; doi:10.1371/journal.pone.0296861)
Supplement: S5 Table — Indicators include mussel presence, log(x+1) species’ CPUE, SPUE, Shannon-Wiener diversity, and Simpson’s diversity. Correlations in bold print were significant after Bonferroni adjustment. The adjusted threshold of significance was p < 0.0002. (DOCX) [file pone.0296861.s005.docx]

**Table S5**. **Spearman correlation coefficients (r) between hydraulic variables and mussel indicators at the mesohabitat scale.** Indicators include mussel presence, log(x+1) species’ CPUE, SPUE, Shannon-Wiener diversity, and Simpson’s diversity. Correlations in bold print were significant after Bonferroni adjustment. The adjusted threshold of significance was p < 0.0002.

| **Mesohabitat scale** | **Discharge** | **Depth (m)** | **Froude number** | **Shear stress (N m^-2^)** | | **Stream power**  **(N-s m^-2^)** |
| --- | --- | --- | --- | --- | --- | --- |
| **Presence** | 0.42 | **0.44** | -0.41 | -0.22 | -0.2 | |
|  | 5.32 | **0.49** | **-0.47** | -0.28 | -0.28 | |
|  | 32.28 | **0.47** | **-0.46** | -0.33 | -0.33 | |
|  | 361.89 | 0.23 | -0.42 | -0.32 | -0.33 | |
| ***L. bracteata* CPUE** | 0.42 | 0.34 | -0.32 | -0.24 | -0.17 | |
|  | 5.32 | 0.37 | -0.38 | -0.24 | -0.21 | |
|  | 32.28 | 0.34 | -0.36 | -0.28 | -0.25 | |
|  | 361.89 | 0.11 | -0.19 | -0.2 | -0.19 | |
| ***U. imbecillis***  **CPUE** | 0.42 | **0.51** | -0.41 | -0.26 | -0.23 | |
|  | 5.32 | **0.55** | **-0.46** | -0.3 | -0.28 | |
|  | 32.28 | **0.56** | **-0.51** | -0.41 | -0.39 | |
|  | 361.89 | 0.26 | -0.38 | -0.29 | -0.28 | |
| **Richness (SPUE)** | 0.42 | -0.03 | 0.01 | 0.18 | 0.13 | |
|  | 5.32 | -0.02 | -0.01 | 0.19 | 0.11 | |
|  | 32.28 | -0.01 | -0.07 | 0.03 | -0.05 | |
|  | 361.89 | -0.07 | -0.31 | -0.2 | -0.24 | |
| **Shannon-Wiener Diversity** | 0.42 | 0.41 | -0.31 | -0.15 | -0.15 | |
|  | 5.32 | 0.4 | -0.31 | -0.13 | -0.15 | |
|  | 32.28 | 0.41 | -0.38 | -0.23 | -0.24 | |
|  | 361.89 | 0.24 | **-0.43** | -0.3 | -0.28 | |
| **Simpson's Diversity** | 0.42 | **0.44** | -0.34 | -0.19 | -0.19 | |
|  | 5.32 | **0.43** | -0.33 | -0.17 | -0.19 | |
|  | 32.28 | **0.44** | -0.39 | -0.24 | -0.24 | |
|  | 361.89 | 0.27 | -0.41 | -0.27 | -0.25 | |
