# Supplementary figures and images for: Go with the flow: Impacts of high and low flow conditions on freshwater mussel assemblages and distribution
Source: PLoS One. 2024 Feb 15;19(2):e0296861. doi: 10.1371/journal.pone.0296861 (PMC10868800; doi:10.1371/journal.pone.0296861)

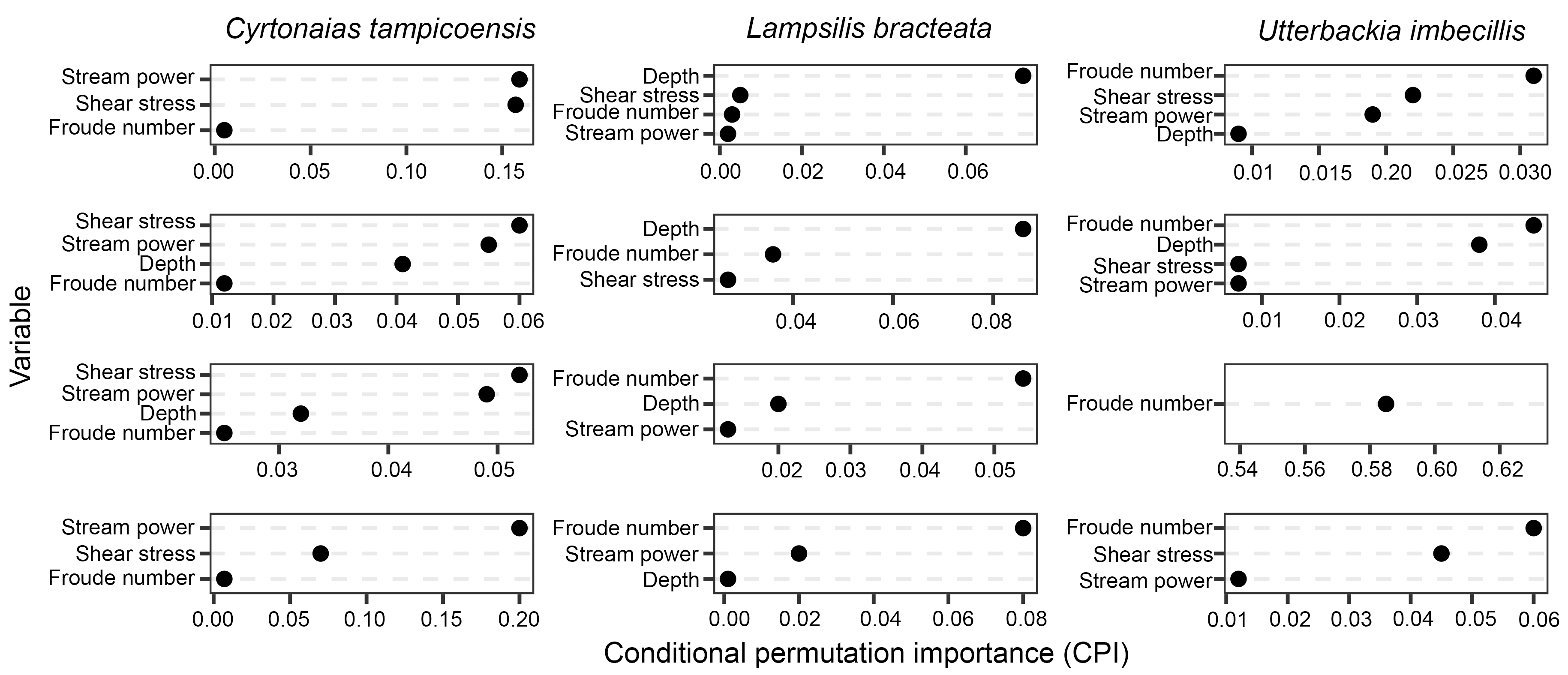

Supplement: S5 Fig — Variable importance was determined using conditional permutation importance (CPI) because some predictor variables were found to be highly correlated [85]. Note: CPI should not be compared across different RF model. (TIF) [file pone.0296861.s010.tif]
