# Supplementary material for: Go with the flow: Impacts of high and low flow conditions on freshwater mussel assemblages and distribution
Source: PLoS One. 2024 Feb 15;19(2):e0296861. doi: 10.1371/journal.pone.0296861 (PMC10868800; doi:10.1371/journal.pone.0296861)
Supplement: S1 Appendix — (DOCX) [file pone.0296861.s011.docx]

**S1 Appendix. Additional methodology for field data collection and HEC-RAS model preparation.**

*Field data collection*

At each of the 200 sites in the 20 km segment of the San Saba River, researchers used tactile and visual search methods for a 0.5 ph timed search to search for freshwater mussels in an approximately 10 m long area spanning the width of the stream (Mitchell, 2020). Survey methods represented a trade-off between increased spatial coverage and survey time but estimates of richness and diversity during surveys were analogous to surveys with higher search efforts during another study in the upper San Saba River (Mitchell et al., 2019). Mussels were counted and identified to the species level before being returned to the riverbed. At each site, mesohabitat type (riffle-run-pool), stream width (m), estimated substrate composition (percent silt, sand, gravel, pebble, cobble, boulder, bedrock), and estimated vegetation density (0, 0-25, 25-50, >75 %) were recorded. Stream depth (m) was measured at three locations across the width of the stream, and mid-channel current velocity (m s^-1^) was measured at 60 % stream depth using an electromagnetic velocity meter (HACH FH950, Hach Company, Loveland, CO 80539 USA). Locations of study sites were recorded using a GPSMap 64 (accuracy of 3-15 m; Garmin International, Inc., Olathe, KS 66062-3426 USA).

*HEC-RAS model*

To prepare a 2D HEC-RAS model for the study segment, discharge data recorded at United States Geological Survey (USGS) streamflow gage 08144500 near Menard, TX, located approximately 15 km downstream of the study segment, were used to inform model building (USGS, 2016). Light detection and ranging (LiDAR) data was collected by the USGS 3-D elevation program (3DEP) between February and April 2018 with 1 m resolution. These data were used to create a terrain in HEC-RAS (Merrick-Surdex, 2018). Flows during this period were comparable to flows during field data collection (0.45-0.71 m^3^s^-1^ versus 0.18-0.51 m^3^s^-1^, respectively). Because inundated areas are represented as a hydro-flattened surface in 3DEP data, a bathymetric surface was interpolated from the data collected at the 200 sites (*i.e*., 1-D cross sections). The hydrosurface was delineated as a shapefile.

One-dimensional cross sections from HEC-RAS were exported to ArcMap (v. 10.8, ESRI, Redlands, CA 92373-8100 USA). The 1-D cross sections were clipped using the hydrosurface shapefile. Next, cross section lengths were calculated in ArcMap using the Geometry Calculator tool. There was some uncertainty in cross section location because of the accuracy of GPS locations, so 1-D cross section lengths were compared to field-measured wetted widths. If widths did not match, cross section locations were moved up or downstream from the field location in an attempt to match wetted widths. If cross section location had to be shifted greater than 1.5 m, field data were flagged and inspected.

Because the discharges and depths from the USGS 3DEP data and field collected data were similar, the initial iteration of bathymetric derivation assumed that field-measured depths (three collected per cross section) were approximately equal to the distance from the hydro-flattened surface to the channel bed. Hence, channels were burned into the LiDAR terrain model following this assumption. The exact locations of field depth measurements were not recorded. For cross sections where all depth measurements were within ± 0.25 m of one another, the average depth was used for the cross section. Cross sections not meeting this criterion used all three depths for delineation.

A raster spanning the width of the channel bed was generated and exploded into points in ArcMap. These points were used to interpolate a channel surface using Inverse-Distance-Weighted (IDW) interpolation. This creates a terrain that fills in areas where backwaters or meanders occur. The interpolated IDW surface was clipped to the hydro-flattened shapefile extent and mosaicked with the initial 3DEP terrain to produce a complete topographic and bathymetric surface.

Landcover in the floodplain was identified using aerial imagery (USDA, 2018). The landcover was categorized as dense woody vegetation, sparse shrub, channel area, or grasslands and delineated (Table S1). Manning’s coefficients were selected based on landcover types (Table S1; Chow, 1959). Manning’s coefficients are roughness coefficients that quantify the amount of resistance the river channel and floodplain produce against flow (Arcement and Schneider, 1989). Resistance to flow is influenced by the physical and environmental characteristics of the channel and the floodplain (Arcement and Schneider, 1989). This includes factors like vegetation or substrate size that may obstruct or slow down water velocity as water passes through or around them. To quantify the uncertainty in the hydraulic conditions at flows higher than the flow during data collection (0.42 m^3^s^-1^), simulations were re-run using the minimum and maximum Manning’s coefficients for selected land uses in the floodplain (Chow, 1959, Table S1). This allowed us to approximate how unrecognized differences in floodplain characteristics may influence hydraulic conditions at higher flows in the absence of discharge data or aerial imagery for flows that result in floodplain inundation. The median value of average site conditions for each site using the minimum and maximum Manning’s coefficients were compared to selected values for the three higher flows to determine if uncertainty in hydraulic conditions may have influenced the results. The 2-D HEC-RAS mesh was set to 3.72 m^2^, with a break line with 7.62 m cell spacing located at the channel center.
